# The Association Between Lifestyle Interventions and Trimethylamine N-Oxide: A Systematic-Narrative Hybrid Literature Review

**DOI:** 10.3390/nu17071280

**Published:** 2025-04-06

**Authors:** Xenophon Theodoridis, Androniki Papaemmanouil, Niki Papageorgiou, Christos Savopoulos, Michail Chourdakis, Areti Triantafyllou

**Affiliations:** 1 Laboratory of Hygiene, Social and Preventive Medicine and Medical Statistics, School of Medicine, Faculty of Health Sciences, Aristotle University of Thessaloniki, 54124 Thessaloniki, Greece; xtheodoridis@auth.gr (X.T.); acpapaem@auth.gr (A.P.); npapaga@auth.gr (N.P.); mhourd@gapps.auth.gr (M.C.); 2 First Propedeutic Department of Internal Medicine, AHEPA University Hospital, Aristotle University of Thessaloniki, 56429 Thessaloniki, Greece; csavvopo@auth.gr

**Keywords:** trimethylamine N-oxide, gut microbiome, dietary interventions, physical activity

## Abstract

Background: Trimethylamine N-oxide (TMAO) is a gut- and food-derived molecule. Elevated TMAO concentrations have been associated with an increased risk of cardiovascular disease (CVD) and all-cause mortality, highlighting its significance as a potential biomarker for adverse health outcomes. Given these associations, it is hypothesized that lifestyle interventions, such as healthy dietary patterns and exercise, could reduce TMAO concentrations. The aim of this systematic–narrative hybrid literature review was to evaluate the relationship between various lifestyle interventions and TMAO. Methods: MEDLINE (via PubMed^®^), Scopus^®^, and grey literature were searched until July 2024 for eligible clinical trials. Case reports, case series, case studies and observational studies were excluded, as well as studies that investigated food products, nutraceuticals, dietary supplements or have been conducted in the pediatric population. Results: In total, 27 studies were included in this review. While some dietary interventions, such as plant-based, high-dairy, very low-calorie ketogenic diet or the Mediterranean diet, were associated with lower TMAO concentrations, others—including high-protein and high-fat diets—were linked to an increase in TMAO concentrations. Studies that incorporated a combination of nutrition and exercise-based intervention presented neutral results. Conclusions: The relationship between dietary interventions and TMAO concentration remains controversial. While certain interventions show promise in reducing TMAO levels, others yield mixed or contradictory outcomes. Further research, including well-structured RCTs, is needed to investigate the aforementioned associations.

## 1. Introduction

Trimethylamine N-oxide (TMAO) consists of a biologically active compound belonging to the class of amine oxides [1]. It is a metabolite that depends on gut microbiota for its production. It is mostly derived from dietary choline, betaine, and L-carnitine, which are initially metabolized into trimethylamine (TMA) by gut microbiota. Subsequently, TMA is converted into TMAO by hepatic flavin monooxygenases (FMOs) [2], and it is then released into circulation [3]. TMAO may also be found naturally in food products such as fish and seafood [4].

TMAO has been considered an atherogenic factor [4], affecting lipid absorption and cholesterol homeostasis and modulating glucose and lipid metabolism [3]. TMAO has been additionally characterized as a precursor of endothelial dysfunction by reducing the expression of endothelial nitric oxide synthase (eNOS), decreasing nitric oxide (NO) production, and increasing levels of endothelin-1 (ET-1) [5]. Moreover, plasma concentrations of TMAO are positively correlated with elevated aortic stiffness and systolic blood pressure in all ages, from healthy young and middle-aged adults to older individuals, independent of cardiovascular disease risk factors [6,7]. As a result, prospective cohort studies have demonstrated that elevated plasma levels of TMAO are predictive of an increased risk for various pathological conditions, including myocardial infarction (MI), stroke, hypertension, atrial fibrillation, heart failure, metabolic syndrome, and mortality [2,8]. Moreover, increased concentrations of TMAO have been associated with chronic kidney disease, type 2 diabetes mellitus, and several types of cancer, such as colorectal and prostate [4] and non-alcoholic fatty liver disease (NAFLD) [3].

Lifestyle interventions or single dietary components, such as a specific food item or nutritional supplement, have been investigated regarding their association with the risk of chronic diseases [9], as well as their effect on TMAO concentrations [10]. Wang and colleagues [11] have shown that long-term red meat consumption has been associated with increased plasma and urine TMAO levels. In a randomized, double-blind, controlled crossover study conducted by Fatani et al. [12], pea hull fiber supplementation did not influence serum TMAO levels in adults undergoing hemodialysis. In the study of Costabile and colleagues [10], dietary patterns rich in long-chain n-3 fatty acids or whole-grain cereals were related to increased TMAO concentration in participants at high cardiometabolic risk. Moreover, physical activity has been linked with improvements in TMAO levels [13]. Physical activity has been especially associated with beneficial effects on gut microbiota, particularly by modifying its composition and diversity.

As the findings of previous studies are contradictory, the aim of this systematic–narrative hybrid literature review was to evaluate and summarize current evidence regarding the relationship between lifestyle interventions and TMAO concentrations.

## 2. Materials and Methods

The methodological approach of this review adhered to Turnbull’s blueprint for a hybrid systematic narrative review [14]. This approach aims to analyze six critical elements: research questions, rationale, literature sources, search parameters, data cleaning, and information synthesis.

### 2.1. Research Question

According to Turnbull’s methodological approach [14], the research question of a systematic–narrative hybrid literature review should be related to search criteria identified in the methodology. This study aims to elucidate the effects of various lifestyle interventions, including modifications in dietary habits, dietary patterns, and physical activity, on trimethylamine N-oxide levels.

The patient-intervention-comparison-outcome (PICO) strategy was utilized to formulate the research question (Table 1). The study included only adult populations, regardless of their health status. The investigation focused solely on lifestyle interventions, such as dietary modifications or patterns or/and physical activity, in comparison to other types of interventions. Furthermore, pre-post studies were also deemed eligible. The primary outcome measured in the study was the TMAO concentrations. Case reports, case series, and case studies were excluded, as well as studies that investigated specific food items, nutraceuticals, pharmacological therapies or dietary supplements. We also excluded observational studies and studies conducted in the pediatric population.

### 2.2. Search Strategy

Eligible studies were identified through MEDLINE (via PubMed^®^) and Scopus^®^, employing text and keywords (MeSH Terms) formulated based on the review’s research question from inception to July 2024 by two independent researchers. Furthermore, grey literature was also searched to minimize bias [14]. Finally, the references of the included studies were examined for any potentially eligible studies.

The search strategy for PubMed was developed and subsequently adapted for use with other databases. The following search terms were used in multiple combinations to support the specified search strings: “TMAO”, “trimethylamine N-oxide”, “lifestyle interventions”, “diet”, “dietary interventions”, “nutrition”, “eating habits”, “exercise”, “physical activity”, “physical exercise”, and “physical activity”. Language restrictions were enforced, including only studies published in English. The full search string is presented in Supplementary Table S1.

### 2.3. Study Selection

All studies were imported into the Rayyan: Reference Manager for Systematic Reviews. Two independent reviewers conducted the screening process. Duplicates were removed, followed by a two-step evaluation of the titles and abstracts and then full texts. A third reviewer resolved any discrepancies concerning study eligibility.

### 2.4. Data Extraction

Two reviewers independently extracted study information into a standardized Excel spreadsheet. Extracted study characteristics included study design, first author’s name, publication year, country, participant characteristics, type of intervention, and statistical data on primary outcomes. Any disagreements were resolved through consensus with a third reviewer. Additionally, the authors were contacted to obtain any missing data relevant to the analysis.

## 3. Results

A total of 4319 articles were retrieved, of which 628 and 136 were screened by title, abstract, and full-text, respectively. After the exclusion of studies that did not meet the eligibility criteria, 27 articles were deemed eligible (Supplementary Figure S1). The eligible studies assessed the effectiveness of dietary (*n* = 22), exercise-based interventions (*n* = 1), or lifestyle interventions (*n* = 4) on TMAO concentrations.

### 3.1. Studies Including Exercise-Based Interventions

Argyridou and colleagues [13] conducted a study using prospective observational data generated from the Walking Away from Type 2 Diabetes trial, including patients at high risk of type 2 diabetes. High risk of type 2 diabetes was defined as impaired glucose tolerance, impaired fasting glycemia, or undiagnosed type 2 diabetes using the Leicester Practice Risk Score. The authors of the study evaluated participants’ physical activity utilizing an accelerometer worn for a week during waking hours. Furthermore, the researchers used food frequency questionnaires to assess participants’ diets at baseline and at 12 months. A total of 438 subjects were included in the study, 65.4% of them being men. The mean and standard deviation (SD) of age and body mass index (BMI) was 63.5 ± 7.3 years and 32.3 ± 5.5 kg/m^2^, respectively. The findings of the study showed that each 30 min or SD difference in moderate to vigorous physical activity was associated with 0.584 μmol/L and 0.456 μmol/L lower TMAO concentrations, respectively, after model adjustment for confounding factors. Moreover, the study found no association between sedentary time and light-intensity physical activity and TMAO concentrations.

### 3.2. Studies Including Nutrition-Based Interventions

#### 3.2.1. Positive Effect on TMAO Concentration

With regard to studies demonstrating a positive effect of the included intervention on TMAO concentrations, Argyridou et al. [15] performed a single-center, interventional, single-group, prospective trial including 23 subjects aged 57.8 ± 10.0 years with overweight or obesity and dysglycemia. None of the participants received any form of diabetic medication. The intervention consisted of an 8-week vegan dietary program, followed by a 4-week unrestricted diet. For the study’s purposes, each participant received a 1.5 h session with a dietitian, in which the food menu and the shopping details were discussed in order to be aligned with personal needs and desires. There were no restrictions on portion sizes, calorie intake, or the macronutrient composition of the diet. On-site visits were performed on the 1st and 8th week of the vegan diet; however, every 10–14 days, telephone follow-ups were conducted in order for the researchers to provide personal support. A 3-day food diary was completed by each subject at baseline and in weeks 1, 8, and 12, and some 24 h recalls were additionally carried out. Plasma TMAO levels decreased (*p* < 0.025), showing a 47% reduction by week 1 and a 40% reduction by week 8 of the vegan diet compared to baseline. By week 12, after returning to the unrestricted diet, TMAO levels returned to baseline. These findings were consistent across different ages, sexes, ethnicities, and weights and were unaffected by any changes in the diet’s macronutrient composition.

The objective of a pilot study conducted by Chiu and colleagues [16] was to evaluate the change in cardiovascular risk factors before and after an intervention with a vegan diet planned by a dietitian. A total of nine individuals with dyslipidemia participated in this study, which lasted for 12 weeks. Among the exclusion criteria were the existence of chronic kidney disease, history of coronary artery disease, ischemic heart disease, heart failure, stroke, or any type of cancer. The vegan diet emphasized brown rice, whole grains, the consumption of 400–600 g of vegetables daily, at least three servings of fruits, beans and soy at least seven servings per day, and at least three servings of nuts daily. The mean age and SD of participants was 58.4 ± 10.0, while the majority of them were women. More than half of patients were classified in the obesity category according to the World Health Organization (WHO) criteria. The findings of the study showed that there was no difference from baseline within the group after 12 weeks of a vegetarian diet on TMAO concentrations. At the individual level, reductions in TMAO were primarily observed in participants who were previously non-vegetarians, particularly those with baseline TMAO > 4 μM. In contrast, TMAO concentrations remained stable in participants who were already vegetarians before the intervention. However, it should be noted that the majority of the vegetarian participants had lower baseline TMAO levels compared to non-vegetarians.

In the study of Diao et al. [17], 120 subjects aged 20–55 years with overweight or obesity were randomized in a 1:1:1 ratio to receive a low-calorie DASH diet, a low-calorie diet, or a control group. The low-calorie diet was consisted of 10–15% of total energy expenditure (TEE) from protein, 55–60% from carbohydrates, and 25–35% from fat, while the DASH diet was consisted of 52–55% carbohydrates, 16–18% protein, and 30% total fat. Subjects participating in this 12-week intervention period did not differ regarding the baseline anthropometric indices, including weight, BMI, and weight circumference. According to the results, TMAO levels did not differ before the intervention (*p* = 0.197) between the three study groups. However, after the intervention, TMAO was found to be diminished in both low-calorie diet (29.70 ± 32.05) and low-calorie DASH diet (21.04 ± 9.03) compared to the control group (38.25 ± 40) (*p* = 0.041). Additionally, differences were observed within low-calorie and low-calorie DASH diet groups when the results before and after the intervention were compared (MD: −10.63, 95% CI: −17.66 to −3.59, *p* = 0.004; MD: −20.04, 95% CI: −27.55 to −12.53, *p* = 0.001 respectively).

In a 16-week randomized, crossover, investigator-blinded, controlled feeding trial, 41 subjects aged 30 to 69 years, with overweight or obesity, consumed either a Mediterranean diet pattern containing approximately 500 g of lean, unprocessed beef or pork per week (Med-Red) or a Mediterranean diet pattern including ~200 g of the aforementioned food (Med-Control) [18]. Both interventions were separated by a washout period of at least 4 weeks, characterized by self-selected eating. The primary differences between the Med-Red and Med-Control dietary patterns were in the quantities of red meat and poultry, along with adjustments in dairy products and grains to ensure macronutrient levels were consistent across both interventions. Participants also had the option to consume 150 mL of dry red wine of their choice each day. The daily macronutrient goals were set to provide 40% of total energy from carbohydrates, 22% from protein, and 40% from fats. Of the daily fat intake, 7% of total energy was aimed to come from saturated fats and 20% from monounsaturated fats. As far as TMAO is concerned [19], a between-group difference (pre- vs. post-intervention) was significant in the Med-Control group (5.2 ± 0.8 vs. 3.1 ± 0.2; *p* = 0.025), whereas in the Med-Red group, no difference was observed (4.4 ± 0.5 vs. 5.0 ± 0.5; *p* = 0.537). Additionally, when the two dietary patterns were compared regarding the post-intervention interaction, a significant difference was detected (post-Med-Control vs. post-Med-Red: *p* < 0.001).

Sun and colleagues [20] conducted an open-label, self-controlled pilot trial aiming to investigate the effect of a fasting diet on endothelial function and vascular injury-related markers in 13 subjects with overweight or obesity aged 25 to 65 years. The total duration of the intervention was 7 days; the first day included an intermediate energy restriction (<800 kcal/day), the next 5 days were characterized by a strict energy restriction (<200 kcal/day), and the last day included a gradual reintroduction of a normal diet (800–1000 kcal/day, consisting of semiliquid or semisolid foods). Furthermore, during the 5-day period, subjects also received a supplement of L-carnitine. All participants were additionally advised to perform an everyday low-intensity physical activity in the form of slow walking. TMAO was one of the characteristics that differed between the participants at the baseline. Following the intervention, the mean concentration of TMAO was reduced compared to the pre fasting diet period (3.96 ± 1.85 vs. 2.73 ± 1.33 μmol/L, *p* = 0.04).

A recently published pilot study aimed to evaluate the effect of the active phase of a very low-calorie ketogenic diet (VLCKD) on hidradenitis suppurativa in twelve treatment-naïve women with overweight or obesity aged 21–54 years old [21]. The prescribed diet attributed less than 800 kcal daily, with a macronutrient composition of 13% from carbohydrates, 43% from proteins, and the remaining 44% coming from fats. To maintain nutritional adequacy throughout the VLCKD, supplements were provided to participants. These included B-complex vitamins, vitamins C and E, essential minerals such as potassium, sodium, magnesium, and calcium, along with omega-3 fatty acids. Compared to the baseline, a reduction in TMAO concentrations was observed (Δ%: −23.67 ± 13.26, *p* < 0.001) after 28 days of the active phase of the VLCKD.

An RCT, including 38 women aged 18–60 years with overweight or obesity, were randomly assigned to follow either a low-dairy or high-dairy diet for 24 weeks in addition to an energy deficit of 500 kcal daily [22]. The macronutrient content of the diets was similar, with 30%, 52%, and 18% of energy coming from fat, carbohydrates, and protein, respectively. The low-dairy diet consisted of 0–1 servings of dairy products daily (<600 mg calcium), while the high-dairy diet included 4–5 dairy products daily (approximately 1200 mg calcium). The researchers reported that the high-dairy diet reduced urinary excretion of TMAO compared to the low-dairy diet, indicating that the high consumption of dairy products is implicated in protein catabolism, energy metabolism, and gut microbial activity.

#### 3.2.2. Negative Effect on TMAO Concentration

Subsequently, studies that reported a negative effect of nutrition-based interventions on TMAO concentrations are presented below.

A secondary analysis of the “Carbohydrate and Related Biomarkers Study” published in 2016 [23]. Briefly, the objective of the study was to assess the extent to which high glycemic load (HGL) and low glycemic load (LGL) diets modify the plasma metabolome, including TMAO, a gut bacterial metabolite. The two diets were balanced to macronutrient distribution and were isocaloric for each participant, with the only difference being observed in the glycemic load of each diet and the fiber content. The LGL diet had half the glycemic load of the HGL, while its fiber content was 55 g/d compared to 28 g/d of the HGL diet. Overall, 19 participants aged 18–45 years were included in the secondary analysis. The metabolites showing the highest fold change between the diets were kynurenate and TMAO, with participants assigned to the LGL diet presenting higher TMAO metabolites compared to participants assigned to the HGL diet. These results remained unchanged after adjusting for weight change, body fat percentage, and fat distribution.

In a crossover trial conducted by Bergeron and colleagues [24], 52 participants aged 44 ± 14 years, with normal blood pressure and overweight or obesity first consumed a baseline diet for two weeks, consisting of 41% of energy from carbohydrates, 40% from fat, and 19% from protein. This dietary program was followed by two-week phases on high- and low-resistant starch (RS) diets, with each phase separated by a two-week washout period. The RS diets were randomly assigned and aligned with either higher (51–53% of energy) or lower carbohydrate intake (39–40% of energy). Baseline TMAO did not differ between those in the lower- and higher-CHO study arms. Fasting plasma TMAO levels were found to be elevated following the high-RS diet compared to the low-RS diet in the lower-carbohydrate treatment group (*p* < 0.01), but this difference was not observed in the higher-carbohydrate treatment group (*p* = 0.41).

A clinical trial aimed to investigate whether a short-term, high-fat diet would increase fasting and postprandial TMAO concentrations due to the effects of a high-fat meal challenge [25]. The study included ten men with a mean age of 22.1 ± 0.5 years and a mean BMI of 22.3 ± 1.0 kg/m^2^. The subjects were weight-stable, physically inactive, and were not receiving antibiotics for at least 6 months prior to the initiation of the study. Additionally, all participants were free of hypertension and diabetes and were required to have a normal lipidemic profile. After compliance with a 2-week eucaloric control diet, all participants consumed a eucaloric high-fat diet (55% fat) for 5 days. The composition of the high-fat diet was 55% fat, 50% of which stemmed from saturated fats, 30% carbohydrates, and 15% protein. To reduce individual differences in habitual dietary intake, the provided diets were controlled. The fasting plasma TMAO concentration did not differ from the baseline following the high-fat diet. The meal challenge did not affect the postprandial TMAO production at baseline. On the contrary, plasma TMAO concentrations showed an increase at 2, 3, and 4 h compared to fasting concentrations after the high-fat diet. Furthermore, during the postprandial period following the high-fat diet, plasma TMAO concentrations were higher at hours 1 to 4 compared with the same time points during the control diet (all *p* < 0.05).

Etherpath’s study [10] included 78 participants with high cardiometabolic risk, who were randomly assigned to one of the following dietary interventions for 8 weeks: (1) control diet, low in long-chain n-3 fatty acids (LCn3) (1.5 g/day) and low in polyphenols (PP) (365 mg/day); (2) high in LCn3 (4 g/day) and low in PP (363 mg/day); (3) high in PP (2903 mg/day) and low in LCn3 (1.4 g/day); and (4) high in PP (2861 mg/day) and high in LCn3 (4 g/day). Salmon (330 g twice a week), dentex, or anchovies (350 g once a week) consisted the primary sources of LCn3 in the interventions, whereas decaffeinated green tea (400 mL, using four tea bags), coffee (4 cups), dark chocolate (25 g), blueberry jam (40 g), extra-virgin olive oil (60 g), and some polyphenol-rich vegetables, including rocket salad (88 g), fennel (200 g), and onions (200 g), provided the necessary amount of PP. Subjects were also instructed to preserve their usual consumption of meat, dairy products, eggs, fish, fruits, vegetables, and fats throughout the entire study period without making any changes. The baseline characteristics of the four groups presented no differences. Regarding the high LCn3 and low PP group, TMAO levels were 7.33 ± 13.93 μmol/L at baseline versus 8.86 ± 7.28 μmol/L at 8 weeks (*p* = 0.640); for the high LCn3 and high PP group, levels were 7.59 ± 7.98 μmol/L at baseline compared to 8.37 ± 4.40 μmol/L at 8 weeks (*p* = 0.713); for the low LCn3 and high PP group, they were 6.65 ± 10.54 μmol/L at baseline versus 5.60 ± 4.00 μmol/L at 8 weeks (*p* = 0.664); and for the low LCn3 and low PP group, levels were 4.97 ± 3.62 μmol/L at baseline compared to 4.86 ± 2.91 μmol/L at 8 weeks (*p* = 0.886). Using a two-factor ANOVA, the changes in TMAO (calculated as 8-week minus baseline) were significant for diets high in LCn3 (*p* = 0.007), while no changes were demonstrated for diets high in PP (*p* = 0.905) or for their interaction (*p* = 0.655).

Mitchell and colleagues [26] conducted an RCT to investigate the effect of dietary protein consumption at twice the current recommended dietary allowance (RDA) in comparison to RDA consumption on TMAO concentrations. The study lasted 10 weeks and included non-smoker males over 70 years old. Eligible participants were randomly assigned to either a diet with a protein consumption of 0.8 g protein/kg body weight/day (RDA group) or a diet with a higher protein intake of 1.6 g/kg body weight per day (2RDA group). The findings of this RCT showed that there was an interaction effect of plasma TMAO (time x diet interaction, *p* = 0.002), which did not change in the RDA group (12.8 ± 9.67 μM vs. 8.05 ± 7.52 μM, *p* = 0.165) and increased in the 2RDA group (from 8.34 ± 4.79 μM to 29.08 ± 31.53 μM, *p* = 0.004). Similarly, an interaction effect of TMAO in urine was observed (time x diet interaction, *p* = 0.007), which did not change in the RDA group (77.4 ± 77 vs. 125.2 ± 134 mmol/mol creatinine, *p* = 0.345) and decreased in the 2RDA group (from 209.8 ± 198 to 77.7 ± 46.2 mmol/mol creatinine, *p* = 0.005). The study concluded that compared to the RDA group, the 2RDA diet group increased circulatory TMAO (*p* = 0.002) but also decreased renal excretion of TMAO (*p* = 0.003).

Park and colleagues [27,28] aimed to investigate the effect of a high-fat diet (Atkins) diet, a Mediterranean diet and a very low-fat diet on TMAO levels, utilizing samples from a previous randomized crossover study, where each isocaloric dietary phase lasted 4 weeks long, followed by a 4-week washout period. Subjects participating in this study were older than 20 years and had a normal lipidemic profile and BMI, as well as absence of metabolic, liver, kidney, or systemic diseases. Compared to the low-fat dietary pattern, the Atkins diet showed higher TMAO levels (3.3 [2.0–4.0] vs. 1.8 [1.2–3.0] mM, *p* = 0.01). Moreover, the Atkins diet was associated with elevated TMAO compared to baseline (3.3 [2.0–4.0] vs. 1.6 [1.1–3.4] mM, *p* = 0.04), although there was no difference in TMAO levels between the Atkins and Mediterranean diet (3.3 [2.0–4.0] vs. 2.6 [1.4–5.0] mM, *p* = 0.7).

A secondary analysis of the Pan-European DiOGenes study, a randomized controlled dietary intervention, was performed by Rasmussen et al. [29], including 109 participants with obesity and absence of diabetes mellitus aged 37–45 years. Subjects were randomized to one of the five intervention groups, which were as follows: (1) low-protein (LP) and low glycemic index (LGI) diet (LP/LGI); (2) LP and high GI diet (LP/HGI); (3) high protein (HP) and LGI diet (HP/LGI); (4) HP and HGI diet (HP/HGI); and (5) control diet, which was structured in agreement with the most recent Danish guidelines, containing a moderate amount of protein. The low-protein diets were formulated to provide 10–15% of energy from protein, while the high-protein diets supplied 23–28% of energy from protein. In terms of carbohydrate content, the high-protein diets were designed to include 45–50% of energy from carbohydrates, whereas the low-protein diets provided 57–62% of energy from carbohydrates. During the 6-month intervention period, 3-day weighed food records were asked to be completed by the participants at baseline, as well as at month 1 and month 6 time points. HP diet has been shown to be associated with an increase in urinary TMAO levels; however, this correlation was described as a “tendency”.

Another RCT aimed to determine the impact of two diets with non-seafood and lean-seafood protein on serum metabolites, including TMAO [30]. The study design was a crossover with two intervention periods lasting 4 weeks, each separated by a 5-week washout period. Prior to the study commencement, the eligible subjects adopted a diet according to the Norwegian nutrition guidelines for a 3-week period. A total of 27 participants started the study, and 19 completed it. The two diets were similar in macronutrient distribution, dietary fiber, and mono-, polyunsaturated and saturated fatty acids. Participants assigned to the non-seafood protein diet consulted to consume chicken, turkey, lean beef, pork, eggs, and dairy products during their lunch and dinner meals, while individuals assigned to the lean-seafood diet consumed pollack, cod, scallops, and saithe with their lunch and dinner meals. To minimize confounding, the researchers added cod liver oil to all non-seafood dinner meals before serving to account for the endogenous marine n-3 fatty acids present in the lean seafood diets. There was an interaction between diet x time x sampling time for the postprandial serum concentration of TMAO (*p* = 0.008). Furthermore, TMAO concentrations were increased after lean seafood compared to a non-seafood diet at all studied postprandial time points.

Stella et al. [31] conducted a metabolic study to investigate the effect of vegetarian, low-meat, and high-meat diets on the metabotype signature of healthy male participants. A total of 12 healthy non-smoking males, aged 25–74 years, and with a body weight ranging from 60 to 81 kg were included in the study. Three different diets were randomly assigned in a cross-over design to study participants. More specifically, the low-meat diet included 60 g/d of meat products, the high-meat diet included 420 g/d of meat products, and the vegetarian diet included 420 g/d of non-meat products. The low-meat, high-meat, and vegetarian diets contained approximately 65 g/d, 143–150 g/d, and 143–150 g/d of protein, respectively. Each dietary regimen was given to participants for 15 days with a washout period of 7 days between each diet. According to the findings, the high-meat diet was associated with increased urinary concentrations of TMAO.

A human-controlled 12-week feeding trial published by Tate et al. [32] included 17 females and 13 males aged 65 years and older. Participants were randomized to consume either 3 oz (85 g; *n* = 15) or 6 oz (170.1 g; *n* = 13) of lean fresh beef as part of a standardized DASH diet. The nutrient composition followed the 2015–2020 dietary guidelines for daily caloric intake for older, sedentary adults, aligning with the DASH eating plan as outlined by the National Heart, Lung, and Blood Institute, National Institutes of Health. Beef portions were evenly divided across breakfast, lunch, and dinner, and a multivitamin supplement suitable for the specific study group was also given on a daily basis. Baseline TMAO levels did not present any difference between the two groups (*p* = 0.98). By week 12, participants who received the 6 oz portion of beef exhibited higher circulating TMAO levels (5.2 nM/mL) compared to those who consumed the 3 oz portion of beef (4.6 nM/mL) (*p* = 0.033). When the outcomes were separated according to the subjects’ sex, no differences were observed regarding TMAO levels, neither at baseline nor at week 12 (*p* = 0.61; *p* = 0.381, respectively). The authors also demonstrated an increase in TMAO levels by 26.5% from week 0 (4.9 nMol/mL) to week 12 (6.2 nMol/mL) (*p* < 0.001), as well as a beef consumption interaction for TMAO (*p* = 0.01).

#### 3.2.3. Neutral Effect on TMAO Concentration

Several studies have reported neutral findings regarding the impact of their interventions on TMAO concentrations.

An RCT was conducted to investigate the research question of whether TMAO concentrations are different in healthy individuals following either a Paleolithic diet or the Australian Guide to Healthy Eating (AGHE) for 4 weeks [33]. In brief, 39 healthy females with a mean age of 47 ± 13 years and a mean BMI of 27 ± 4 kg/m^2^ were randomized into the two intervention groups. Three days of weighed food records were used to assess dietary intake pre- and post-intervention. After the 4-week intervention, there was no difference between the two groups regarding the TMAO concentrations, despite the greater consumption of red meat and egg in the Paleolithic diet group.

The Healthy Eating Study for Colon Cancer Prevention is an RCT conducted in the US, where participants were assigned to either the Mediterranean diet group or to the Healthy Eating diet (HED) according to the dietary recommendations for Healthy People 2020 for six months [34]. Overall, 120 healthy subjects with a BMI ranging from 18.5 to 35 kg/m^2^ and at risk of colon cancer were enrolled in the study. Of them, 93 participants, 52 ± 12 years old and with a mean BMI of 27 ± 3.7 kg/m^2^, completed the six months study duration. The macronutrient composition of the HED consisted of <35% fat, <10% saturated fat, two and three servings daily of fruits and vegetables, respectively, as well as three servings per day of whole grains. On the other hand, the MD characteristics were 7–9 servings of fruits and vegetables daily, consumption of omega-3-rich foods at least two times weekly, and at least three servings daily of whole grains. Furthermore, this dietary regimen aimed to a target 1:2:5 ratio for polyunsaturated fatty acid: saturated fatty acid: monounsaturated fatty acid. The fasting serum concentrations of TMAO were measured at baseline and after 6 months of intervention. No changes were observed in the serum concentrations of TMA precursors or TMAO in either treatment group following the intervention. Furthermore, the ratios of TMAO to its precursors, which indicate the conversion of TMA to TMAO, remained consistent across both treatment groups.

Another RCT, the POUNDS Lost trial [35], included 811 participants with overweight or obesity. Of the total number of participants, 510 had blood samples and TMAO measurements and were included in this secondary analysis [36]. Individuals were assigned to four low calorie diets (LCDs) with different macronutrient distributions. The first group received a low-fat average-protein diet (20% fat, 15% protein, and 65% carbohydrates), the second group a low-fat high-protein diet (20% fat, 25% protein, and 55% carbohydrates), the third arm, a high-fat average-protein diet (40% fats, 15% protein, and 45% carbohydrates), and the last group received a high-fat high-protein diet (40% fat, 25% protein, and 35% carbohydrates). Over the 6 months following diet interventions, interindividual variability in the changes in TMAO levels (ranging from −24.4 to 22.4 μmol/L), with a median change of 0 μmol/L (IQR: −1.2 to 1.3 μmol/L), was observed. Additionally, an absence of difference was demonstrated in mean ΔTMAO values across the various diets with differing levels of fat, protein, or carbohydrates.

An interventional study recruited 43 generally healthy omnivorous individuals and assigned them to a regular diet in addition to vegetables (control diet) or a fasting-mimicking diet (FMD) for five days [37]. In short, subjects in the FMD group followed a 5-day LCD that provided 34–54% of regular caloric intake. The participants were recommended to consume mainly complex carbohydrates and unsaturated fat and to avoid protein consumption (Cho: 40–45%, Unsaturated fat: 45–50%, and Pro: 10–15%). Individuals assigned to the regular diet were instructed to include four servings of vegetables in their standard diet. FMD results in a twofold decrease in plasma TMAO concentrations, where there was no observed difference in TMAO levels with the control diet. More specifically, there was a decrease in TMAO concentrations in 8 out of 19 participants assigned to the control diet; however, the remaining 11 individuals presented an increase in TMAO concentrations. On the contrary, most participants (75%) in the intervention group reduced their plasma TMAO concentrations.

A 6-day controlled feeding trial aimed to assess the serum and urine concentrations of TMAO between patients with chronic kidney disease (CKD) and matched adults without CKD. A total of 16 adults, 8 in each group, were recruited for this study [38]. Individuals consumed a 3-day cycle menu with consistent nutrient content for macronutrients and some micronutrients, such as phosphorus, calcium, and salt. The protein content came primarily from animal products (70%), and the remaining 30% was provided by plant-based foods, while the insoluble and soluble fiber content of the diet was 72 and 28%, respectively. Serum TMAO concentrations were not different between the two groups; however, the study indicated that urine TMAO concentrations were higher in the CKD population than in their matched controls.

### 3.3. Studies Including Lifestyle Interventions

A lifestyle-based “Immersion Program” [39] was conducted in 2019, including 73 adults, with a mean age of 46.89 ± 12.38 years and a mean BMI of 31.14 ± 8.83 kg/m^2^. The trial consisted of a 1-week daily nutrition training, aiming to eliminate sugar, salt and oil and to focus on the consumption of plant-based foods, physical activity, and stress management. No energy or quantity limitation was applied. In total, the program consisted of 15 h of dietary education, 5 h of exercise-focused training, 2 h of stress management coaching, and 4 h of cooking sessions. TMAO levels remained unaffected after this short-period intervention (*p* = 0.7488).

A clinical trial published in 2023 aimed to evaluate whether an LCD in addition to interval exercise (LCD + INT) is more effective in improving TMAO concentrations compared to an LCD alone [40]. Overall, 23 women with obesity and a sedentary lifestyle were included in the study. The exclusion criteria of the study included physically active participants (>60 min/week), pregnant or nursing women, or individuals on medication known to affect glucose metabolism and/or blood pressure, smokers within the previous 2 years or subjects with an unstable weight for the last six months. Women in the LCD group were consuming 1000–1200 kcal/d based on diets recommended for adults with obesity who will undergo bariatric surgery. To evaluate participants’ compliance with the provided diet, researchers used 13-day food records. The subjects assigned to the LCD + INT group also completed 12 INT sessions under supervision over 13 days. The duration of the exercise was gradually increased, starting from 30–45 min in the first two days and reaching 60 min of exercise thereafter. The exercise sessions involved participants starting with a 3 min warm-up cycling at 50% peak heart rate (HRpeak), followed by alternating 3 min intervals of cycling at 90% and 50% HRpeak throughout the 60 min duration. The findings of the study indicated that there was no difference in TMAO concentrations following either intervention. However, higher baseline TMAO concentrations were associated with greater reduction following either intervention. Moreover, increased fasting pulse pressure amplification was associated with decreased TMAO.

Erickson et al. [41] conducted and published a post-hoc analysis of a randomized controlled trial in order to assess the effects of two different lifestyle interventions on cardiometabolic risk factors. The participants were randomly assigned to either a 12-week eucaloric diet in addition to exercise training or to a hypocaloric diet combined with exercise training. The included subjects were weight-stable, physically inactive, and did not receive any medication known to have an effect on outcomes of interest. The exercise training was similar for both groups, occurring 5 days a week for a duration of 50–60 min on either a treadmill or cycle ergometer. Initial maximal heart rate (HRmax) was set between 60–65%, while it was progressively increased in order for participants to achieve an intensity of 80–85% at week 4. An exercise physiologist supervised all the exercise training. Participants in the hypocaloric diet were counseled to reduce their daily energy intake by 500 kcal. A weekly session with a dietitian was provided to participants so they can achieve the 500-kcal deficit target. Adherence to the diet was evaluated using 3-day food recalls. At baseline, there was no difference in TMAO concentrations between the two study arms. After the intervention, there was no difference in the absolute change of TMAO concentrations within and between the groups. However, the average percentage change in TMAO was different between the groups (EU: 32 ± 0.6% vs. HYPO: −31 ± 0.4%, *p* = 0.04).

An experimental study aimed to evaluate whether TMAO concentrations are decreased before and after the consumption of a high-fat diet (HFD) between males following an endurance physical activity program and sedentary ones [42]. A total of 24 males, 17 endurance-trained and seven sedentary, ranging from 18–40 years, were included in the study. To minimize interindividual variability of habitual diet intake, the assigned diets were controlled and standardized. Firstly, participants followed an eucaloric diet made up of 55% carbohydrates, 30% total fat, and <10% saturated fat for a period of 10 days. After this period, the included subjects completed a high-fat challenge meal testing containing two breakfast sandwiches with sausage, egg, and cheese. Following, individuals adopted an HFD for 5 days and then a second high-fat challenge session. Blood samples were taken from all participants at baseline, after an overnight fast, and each hour for four hours after the intake of the high-fat challenge meal. The findings of this feeding study showed that there was no difference between the two groups at baseline, fasting, or postprandial TMAO concentrations.

A summary of all the studies mentioned can be found in Table 2.

## 4. Discussion

This systematic–narrative hybrid literature review indicated that current evidence highlights a complex relationship between dietary interventions and TMAO concentrations, influenced by multiple factors, including dietary composition, individual variability, and baseline TMAO concentrations. Moderate-to-vigorous physical activity (MVPA) seems to have a positive but modest effect on TMAO concentrations.

Increased TMAO has been established as a potential novel marker and risk factor for cardiovascular disease and other health-related outcomes [43,44]. For example, studies show that elevated TMAO concentrations lead to platelet hyperactivity, increased risk for diabetes and colorectal cancer development, prolongation of angiotensin II effects, and decrease of fatty acids β-oxidation in the heart [45]. As a diagnostic marker, it is associated with diabetes risk and complications related to diabetes, mortality in patients with coronary artery disease or pneumonia, and clinical events in patients with chronic kidney disease [45].

Lifestyle interventions, particularly diet and physical activity, may affect TMA concentrations and its metabolic byproduct, namely TMAO, by altering dietary precursors of TMA and gut microbiota. Choline, l-carnitine, and betaine are the main dietary precursors of TMA. However, the biological pathway in which dietary components influence TMAO concentrations is intricate, shaped by genetic and microbial factors that regulate the conversion of dietary precursors into TMAO [46,47]. The relationships between TMAO, lifestyle factors (such as diet and exercise), and disease are complex, making it challenging to draw definitive conclusions given the current scientific understanding.

Dietary alterations such as the reduction of fish, red meat, and eggs consumption is postulated to lead to a decrease in TMAO concentrations, even though according to systematic reviews and meta-analysis, fish intake is associated with decreased CVD risk in patients at risk [48] and eggs consumption is not associated with CVD risk [49]. Furthermore, compliance with a plant-based diet may also have a favorable effect on TMAO concentrations, as those diets are typically lower in TMA precursors [50].

Even though physical activity could affect gut microbiome diversity and distribution [51,52], the evidence regarding the effects of physical activity on TMAO concentrations remains inconclusive. There is also an observation that the beneficial effect of physical activity on TMAO concentrations is not acute, and long-term adoption of exercise sessions is needed [41]. Physical activity enhances insulin sensitivity, reduces insulin resistance [53], and has a protective role against chronic inflammation-associated diseases, which may be attributed to the anti-inflammatory effect of regular exercise [54]. These factors may, in turn, lead to altered gut microbiota diversity and distribution and gut dysbiosis [55]. Additionally, exercise mitigates risk factors that may be associated with elevated TMAO concentrations, such as dyslipidemia [56] and hypertension [57], even though the direct effects on TMA production remain less clear.

It should be noted that TMAO plasma concentrations present high intraindividual variability [58], are influenced by gut microbiota composition, which is affected by non-modifiable factors such as age [59], sex [60], and ethnicity [61], and are strongly related to kidney function [62,63]. These confounding factors may affect the relationship between lifestyle interventions and TMAO concentrations, and future studies should aim to minimize their impact by utilizing rigorous methodologies and prioritizing randomized controlled trials as the main study design.

This systematic–narrative hybrid review has provided valuable insights; however, certain limitations must be acknowledged. First, the heterogeneity in dietary and physical activity interventions, intervention durations, and assessment methods may impact the generalizability of the findings. Second, several studies included relatively small sample sizes, which raises concerns regarding the robustness of the results. Furthermore, as previously mentioned, some studies may not consider key confounding factors and appropriate methods for addressing them, potentially compromising the quality of the evidence.

## 5. Conclusions

TMAO represents a promising yet complex biomarker with significant implications for cardiovascular health and other metabolic diseases. While dietary adjustments, particularly a shift towards plant-based diets and sustained physical activity, show potential for mitigating elevated TMAO concentrations, current evidence remains inconclusive and complicated by confounding variables such as gut microbiota composition, kidney function, and demographic factors. Future research, particularly well-designed RCTs, is essential to unravel the intricate mechanisms governing TMAO production and its health outcomes; these could allow focused interventions to improve clinical outcomes.

## Figures and Tables

**Table 1:** PICO Strategy.

PICO Acronym Criteria	PICO Items Relevant to Eligibility Criteria
(P) Population	Adults
(I) Intervention	Any lifestyle intervention, including dietary modifications and/or physical activity
(C) Comparator	Any lifestyle intervention including control diet, other lifestyle intervention
(O) Outcomes	TMAO concentrations in blood or urine

TMAO: Trimethylamine N-oxide.

**Table 2:** Study and clinical characteristics of the included studies.

Study ID	Study Design	Population	Interventions	Findings
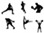	Studies, including exercise-based intervention			
Argyridou et al., 2020 [13]	Prospective observational data from a cluster RCT	P (Ν = 483) at high risk of T2DM, aged 63.5 ± 7.3 years	Arm 1: “Walking Away from Type 2 Diabetes” structured education program (increase of physical activity levels up to 3000 step/day over baseline levels) Arm 2: Control conditions	30 min or SD difference in MVPA → 0.584 μmol/L and 0.456 μmol/L ↓ TMAO concentrations
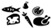	Studies including nutrition-based intervention			
Argyridou et al., 2021 [15]	Single-center, interventional, single-group, prospective trial	P (N = 23) aged 57.8 ± 10.0 years with overweight and dysglycemia, or obesity	8-week VD, followed by a 4-week UD	VD: ↓ TMAO levels by week 1, ↓ by week 8 UD: TMAO levels returned to baseline
Chiu et al., 2022 [16]	One-arm pilot intervention study	P (N = 9) with dyslipidemia, aged 58.4 ± 10.0 years	Vegan diet	TMAO ↓ in previously non-vegetarians P & constant for vegetarians
Diao et al., 2024 [17]	RCT	P (N = 120) with overweight or obesity, aged 41.68 ± 9.66, 44.12 ± 10.22, 41.73 ± 9.66 years in each arm, respectively	Arm 1: LCD DASH Arm 2: LCD Arm 3: Control diet	↓ TMAO in LCD and LCD DASH compared to control
Krishnan et al., 2022 [19]	Randomized, crossover, investigator-blinded, controlled feeding trial	P (N = 39) with overweight or obesity, aged between 30 and 69 years	Arm 1: Med-Red Arm 2: Med-Control	Med-Control ↓ TMAO concentrations compared to Med-Red
Sun et al., 2020 [20]	Open-label, self-controlled pilot trial	P (N = 13) with overweight or obesity, aged 43.54 ± 10.60 years	7-day fasting diet 1st day: <800 kcal/day 2nd–6th day: <200 kcal/day 7th day: 800–1000 kcal/day	↓ TMAO after the intervention
Verde et al., 2024 [21]	Pilot study	Treatment-naïve women (N = 12) with overweight or obesity, aged 21 to 54 years	VLCKD	↓ in TMAO concentrations
Zheng et al., 2016 [22]	RCT	Women (N = 38) with overweight or obesity, aged 45.2 ± 2.9, 41.3 ± 2.7 years in each arm, respectively	Arm 1: LD diet + ED of 500 kcal/day Arm 2: HD diet + ED of 500 kcal/day	HD diet → ↓ urinary TMAO excretion
Barton et al., 2015 [23]	Randomized, controlled, crossover feeding trial	Healthy individuals (N = 19; men: 31.3 ± 9.2 years, women: 31.9 ± 9.5 years)	Arm 1: HGL Arm 2: LGL	TMAO showed the highest fold change between the diets TMAO 37% ↑ at the end of the LGL compared to the HGL intervention
Bergeron et al., 2016 [24]	Crossover trial	P (N = 52) with normal BP and overweight or obesity, aged 44 ± 14 years	Baseline diet: 41% carbohydrates, 40% fat, 19% protein Arm 1: High-RS, HC diet Arm 2: High-RS, LC diet Arm 3: Low-RS, HC diet Arm 4: Low-RS, LC diet	↑ TMAO levels following the high-RS diet in the lower-carbohydrate treatment group (*p* < 0.01) ⟷ in the higher-carbohydrate treatment group (*p* = 0.41)
Boutagy et al., 2015 [25]	Controlled feeding trial	Hypertension and diabetes-free men (N = 10) aged 22.1 ± 0.5 years, with a normal lipidemic profile	2-week EU control diet followed by a 5-day EU HFD	↑ plasma TMAO following the high-fat diet
Costabile et al., 2021 [10]	RCT	P (N = 78) with high cardiometabolic risk, aged 54 ± 9, 56 ± 8, 53 ± 9, 55 ± 9 years in each arm, respectively	Arm 1: control diet, low LCn3 + low PP Arm 2: high in LCn3 + low PP Arm 3: high PP + low LCn3 Arm 4: high PP + high LCn3	Changes (↑) in TMAO for diets high in LCn3 (*p* = 0.007), no changes for diets high in PP (*p* = 0.905) or for their interaction (*p* = 0.655)
Mitchell et al., 2019 [26]	RCT	Non-smoker males (N = 29), aged 74.7 ± 3.9, 73.7 ± 3.3 years in each arm, respectively	Arm 1: RDA group (0.8 g protein/kg body weight/day) Arm 2: 2RDA group (1.6 g/kg body weight per day)	Interaction effect of plasma TMAO (time x diet interaction, *p* = 0.002): RDA group ⟷ 2RDA group ↑ Interaction effect of TMAO in urine (time x diet interaction, *p* = 0.007): RDA group: ⟷ 2RDA group: ↑
Park et al., 2019 [27]	Randomized crossover study	P (N = 14) with normal lipidemic profile and BMI, absence of metabolic, liver, kidney, or systemic diseases, aged 30.6 ± 9.6 years (mean age refers to N = 18)	Arm 1: HF (Atkins) diet Arm 2: MD Arm 3: VLF diet	Atkins: ↑ TMAO compared to VLF and baseline ⟷ TMAO between Atkins and MD
Rasmussen et al., 2012 [29]	Randomized controlled dietary intervention	P (N = 77) with obesity and absence of DM (HP diet: 43.9 ± 4.9 years, LP diet: 42 ± 5.1 years)	Arm 1: LP/LGI diet Arm 2: LP/HGI diet Arm 3: HP/LGI diet Arm 4: HP/HGI diet Arm 5: Control diet	HP: ↑ urinary TMAO levels (tendency)
Schmedes et al., 2018 [30]	RCT	Healthy adults (N = 20), aged 50.6 ± 3.4 years	Arm 1: Non-seafood diet Arm 2: Lean-seafood diet	Diet x time x sampling time interaction for the postprandial serum concentration of TMAO (*p* = 0.008) ↑ TMAO concentrations after lean-seafood
Stella et al., 2006 [31]	Randomized crossover design	Healthy male P (N = 12), aged between 25 and 74 years	Arm 1: Vegetarian diet Arm 2: Low meat diet Arm 3: High meat diet	High-meat diet → ↑ urinary TMAO concentrations
Genoni et al., 2019 [33]	RCT	Healthy individuals (N = 39), aged 47 ± 13 years	Arm 1: Paleolithic diet Arm 2: AGHE	⟷ TMAO concentrations between groups
Griffin et al., 2019 [34]	Data from a previous RCT	Healthy subjects (N = 115 at baseline, 90 post-intervention) with BMI 18.5 to 35 kg/m^2^ and at risk of colon cancer, aged 52 ± 12 years	Arm 1: MD group Arm 2: HED	No impact on TMAO concentrations
Heianza et al., 2018 [36]	RCT	P (N = 510) with overweight or obesity, aged 51.5 ± 9 years	Arm 1: low-fat average-protein diet Arm 2: low-fat high-protein diet Arm 3: high-fat average-protein diet Arm 4: high-fat high-protein diet	⟷ in mean ΔTMAO across the diets
Tate et al., 2023 [32]	Human-controlled feeding trial	P (N = 28) aged 65 years and older (65–84 years)	Arm 1: 3 oz beef as part of DASH diet Arm 2: 6 oz beef as part of DASH diet	6 oz-portion ↑ TMAO compared to 3 oz-portion of beef ↑ TMAO from week 0 to week 12 Beef consumption interaction for TMAO (*p* = 0.01)
Videja et al., 2022 [37]	Interventional study	Healthy omnivorous individuals (N = 43; FMD: 39 ± 2 years, CD: 37 ± 3 years)	Arm1: FMD Arm 2: CD	FMD: 8 out of 19 P → ↓ in TMAO concentrations, 11 P → ↑ in TMAO concentrations CD: ⟷ TMAO concentrations
Wiese et al., 2024 [38]	Secondary analysis of a parallel-arm, controlled feeding study	P (N = 16) with CKD (mean age = 57 ± 14 years) and matched adults without CKD (mean age = 52 ± 13 years)	3-day cycle menu with consistent nutrient content for macro- and some micronutrients	⟷ TMAO concentrations between groups Urine TMAO concentrations ↑ in CKD P
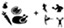	Studies including a combination of nutrition and exercise-based intervention			
Ahrens et al., 2021 [39]	Lifestyle-based trial	Adults (N = 73) aged 46.89 ± 12.38 years	1-week daily nutrition training program (constituted three meals/day, participation in daily exercise and yoga classes)	⟷ TMAO after the intervention
Battillo et al., 2023 [40]	Randomized clinical trial	Women (N = 23) with obesity and sedentary lifestyle, aged 48.4 ± 2.4 years	Arm 1: LCD group Arm 2: LCD + INT group	⟷ TMAO concentrations between interventions ↑ baseline TMAO concentrations → ↑ reduction following either intervention
Erickson et al., 2019 [41]	Post-hoc analysis of an RCT	Weight stable, physically inactive P (N = 16), aged 66.1 ± 4.4 years	Arm 1: EU diet + exercise training Arm 2: HYPO diet + exercise training	⟷ TMAO concentrations within and between the groups
Steele et al., 2021 [42]	Experimental study	Endurance-trained or sedentary individuals (N = 24; endurance trained: 22 ± 2 years, sedentary: 23 ± 3 years)	Arm 1: HFD + endurance physical activity program Arm 2: HFD + sedentary lifestyle	⟷ TMAO concentrations between groups

↑—higher; ↓—lower; ⟷—no change. AGHE—Australian Guide to Healthy Eating; BMI—Body Mass Index; BP—Blood Pressure; CD—Control Diet; CKD—Chronic Kidney Disease; DASH—Dietary Approaches to Stop Hypertension; DM—Diabetes Mellitus; ED—Energy Deficit; EU—Eucaloric; FMD—Fasting Mimicking Diet; HC—High Carbohydrate; HD—High Dairy; HED—Healthy Eating Diet; HFD—High-Fat Diet; HGI—High Glycemc Index; HGL—High Glycemic Load; HP—High Protein; HYPO—Hypocaloric; INT—Interval Exercise; IQR—Interquartile Range; LC—Low Carbohydrate; LD—Low Dairy; LCD: Low-Calorie Diet; LCn3—Long-Chain n-3 fatty acids; LGL—Low Glycemic Load; LP—Low Protein; MD—Mediterranean Diet; MVPA—Moderate-to-Vigorous Physical Activity; P—Participants; PP—Polyphenols; RCT—Randomized Controlled Trial; RDA—Recommended Dietary Allowances; RS—Resistant Starch; T2DM—Type 2 Diabetes Mellitus; TMA—Trimethylamine; TMAO—Trimethylamine N-oxide; UD—Unrestricted Diet; VD—Vegan Diet; VLCKD—Very Low-Calorie Ketogenic Diet; VLF—Very Low-Fat.

## Data Availability

No new data were created or analyzed in this study. Data sharing is not applicable to this article.

## Supplementary Materials

The following supporting information can be downloaded at: https://www.mdpi.com/article/10.3390/nu17071280/s1, Figure S1: Flowchart of the eligibility process; Table S1: Search strategy for identifying studies on PubMed.

## References

[1] Shanmugham M., Bellanger S., Leo C.H. (2023). Gut-Derived Metabolite, Trimethylamine-N-Oxide (TMAO) in Cardio-Metabolic Diseases: Detection, Mechanism, and Potential Therapeutics. Pharmaceuticals.

[2] Yang S., Li X., Yang F., Zhao R., Pan X., Liang J., Tian L., Li X., Liu L., Xing Y. (2019). Gut Microbiota-Dependent Marker TMAO in Promoting Cardiovascular Disease: Inflammation Mechanism, Clinical Prognostic, and Potential as a Therapeutic Target. Front. Pharmacol..

[3] Chen Y.M., Liu Y., Zhou R.F., Chen X.L., Wang C., Tan X.Y., Wang L.J., Zheng R.D., Zhang H.W., Ling W.H. (2016). Associations of Gut-Flora-Dependent Metabolite Trimethylamine-N-Oxide, Betaine and Choline with Non-Alcoholic Fatty Liver Disease in Adults. Sci. Rep..

[4] Barrea L., Annunziata G., Muscogiuri G., Laudisio D., Di Somma C., Maisto M., Tenore G.C., Colao A., Savastano S. (2019). Trimethylamine N-Oxide, Mediterranean Diet, and Nutrition in Healthy, Normal-Weight Adults: Also a Matter of Sex?. Nutrition.

[5] Iglesias-Carres L., Hughes M.D., Steele C.N., Ponder M.A., Davy K.P., Neilson A.P. (2021). Use of Dietary Phytochemicals for Inhibition of Trimethylamine N-Oxide Formation. J. Nutr. Biochem..

[6] Brunt V.E., Casso A.G., Gioscia-Ryan R.A., Sapinsley Z.J., Ziemba B.P., Clayton Z.S., Bazzoni A.E., Vandongen N.S., Richey J.J., Hutton D.A. (2021). Gut Microbiome-Derived Metabolite Trimethylamine N-Oxide Induces Aortic Stiffening and Increases Systolic Blood Pressure With Aging in Mice and Humans. Hypertension.

[7] Casso A.G., Gioscia-Ryan R.A., Sapinsley Z.J., Van Dongen N.S., Bazzoni A.E., Neilson A.P., Zigler M.C., Davy K.P., Seals D.R., Brunt V.E. (2020). YI 1.4 Increases in Circulating Trimethylamine-N-Oxide Contribute to the Development of Age-Related Aortic Stiffness in Humans and Mice. Artery Res..

[8] Mudimela S., Vishwanath N.K., Pillai A., Morales R., Marrelli S.P., Barichello T., Giridharan V.V. (2022). Clinical Significance and Potential Role of Trimethylamine N-Oxide in Neurological and Neuropsychiatric Disorders. Drug Discov. Today.

[9] Xiao Y.L., Gong Y., Qi Y.J., Shao Z.M., Jiang Y.Z. (2024). Effects of Dietary Intervention on Human Diseases: Molecular Mechanisms and Therapeutic Potential. Signal Transduct. Target. Ther..

[10] Costabile G., Vetrani C., Bozzetto L., Giacco R., Bresciani L., Del Rio D., Vitale M., Della Pepa G., Brighenti F., Riccardi G. (2021). Plasma TMAO Increase after Healthy Diets: Results from 2 Randomized Controlled Trials with Dietary Fish, Polyphenols, and Whole-Grain Cereals. Am. J. Clin. Nutr..

[11] Wang Z., Bergeron N., Levison B.S., Li X.S., Chiu S., Xun J., Koeth R.A., Lin L., Wu Y., Tang W.H.W. (2019). Impact of Chronic Dietary Red Meat, White Meat, or Non-Meat Protein on Trimethylamine N-Oxide Metabolism and Renal Excretion in Healthy Men and Women. Eur. Heart J..

[12] Fatani A.M.N., Suh J.H., Auger J., Alabasi K.M., Wang Y., Segal M.S., Dahl W.J. (2023). Pea Hull Fiber Supplementation Does Not Modulate Uremic Metabolites in Adults Receiving Hemodialysis: A Randomized, Double-Blind, Controlled Trial. Front. Nutr..

[13] Argyridou S., Bernieh D., Henson J., Edwardson C.L., Davies M.J., Khunti K., Suzuki T., Yates T. (2020). Associations between Physical Activity and Trimethylamine N-Oxide in Those at Risk of Type 2 Diabetes. BMJ Open Diabetes Res. Care.

[14] Turnbull D., Chugh R., Luck J. (2023). Systematic-Narrative Hybrid Literature Review: A Strategy for Integrating a Concise Methodology into a Manuscript. Soc. Sci. Humanit. Open.

[15] Argyridou S., Davies M.J., Biddle G.J.H., Bernieh D., Suzuki T., Dawkins N.P., Rowlands A.V., Khunti K., Smith A.C., Yates T. (2021). Evaluation of an 8-Week Vegan Diet on Plasma Trimethylamine-N-Oxide and Postchallenge Glucose in Adults with Dysglycemia or Obesity. J. Nutr..

[16] Chiu T.H.T., Kao Y.C., Wang L.Y., Chang H.R., Lin C.L. (2022). A Dietitian-Led Vegan Program May Improve GlycA, and Other Novel and Traditional Cardiometabolic Risk Factors in Patients With Dyslipidemia: A Pilot Study. Front. Nutr..

[17] Diao Z., Molludi J., Latef Fateh H., Moradi S. (2024). Comparison of the Low-Calorie DASH Diet and a Low-Calorie Diet on Serum TMAO Concentrations and Gut Microbiota Composition of Adults with Overweight/Obesity: A Randomized Control Trial. Int. J. Food Sci. Nutr..

[18] O’Connor L.E., Paddon-Jones D., Wright A.J., Campbell W.W. (2018). A Mediterranean-Style Eating Pattern with Lean, Unprocessed Red Meat Has Cardiometabolic Benefits for Adults Who Are Overweight or Obese in a Randomized, Crossover, Controlled Feeding Trial. Am. J. Clin. Nutr..

[19] Krishnan S., O’Connor L.E., Wang Y., Gertz E.R., Campbell W.W., Bennett B.J. (2021). Adopting a Mediterranean-Style Eating Pattern with Low, but Not Moderate, Unprocessed, Lean Red Meat Intake Reduces Fasting Serum Trimethylamine N-Oxide (TMAO) in Adults Who Are Overweight or Obese. Br. J. Nutr..

[20] Sun J., Zhang T., Zhang L., Ke B., Qin J. (2020). Fasting Therapy Contributes to the Improvement of Endothelial Function and Decline in Vascular Injury-Related Markers in Overweight and Obese Individuals via Activating Autophagy of Endothelial Progenitor Cells. Evid. Based Complement. Alternat Med..

[21] Verde L., Cacciapuoti S., Caiazzo G., Megna M., Martora F., Cavaliere A., Mattera M., Maisto M., Tenore G.C., Colao A. (2024). Very Low-Calorie Ketogenic Diet (VLCKD) in the Management of Hidradenitis Suppurativa (Acne Inversa): An Effective and Safe Tool for Improvement of the Clinical Severity of Disease. Results of a Pilot Study. J. Transl. Med..

[22] Zheng H., Lorenzen J.K., Astrup A., Larsen L.H., Yde C.C., Clausen M.R., Bertram H.C. (2016). Metabolic Effects of a 24-Week Energy-Restricted Intervention Combined with Low or High Dairy Intake in Overweight Women: An NMR-Based Metabolomics Investigation. Nutrients.

[23] Barton S., Navarro S.L., Buas M.F., Schwarz Y., Gu H., Djukovic D., Raftery D., Kratz M., Neuhouser M.L., Lampe J.W. (2015). Targeted Plasma Metabolome Response to Variations in Dietary Glycemic Load in a Randomized, Controlled, Crossover Feeding Trial in Healthy Adults. Food Funct..

[24] Bergeron N., Williams P.T., Lamendella R., Faghihnia N., Grube A., Li X., Wang Z., Knight R., Jansson J.K., Hazen S.L. (2016). Diets High in Resistant Starch Increase Plasma Levels of Trimethylamine-N-Oxide, a Gut Microbiome Metabolite Associated with CVD Risk. Br. J. Nutr..

[25] Boutagy N.E., Neilson A.P., Osterberg K.L., Smithson A.T., Englund T.R., Davy B.M., Hulver M.W., Davy K.P. (2015). Short-Term High-Fat Diet Increases Postprandial Trimethylamine-N-Oxide in Humans. Nutr. Res..

[26] Mitchell S.M., Milan A.M., Mitchell C.J., Gillies N.A., D’souza R.F., Zeng N., Ramzan F., Sharma P., Knowles S.O., Roy N.C. (2019). Protein Intake at Twice the RDA in Older Men Increases Circulatory Concentrations of the Microbiome Metabolite Trimethylamine-N-Oxide (TMAO). Nutrients.

[27] Park J.E., Miller M., Rhyne J., Wang Z., Hazen S.L. (2019). Differential Effect of Short-Term Popular Diets on TMAO and Other Cardio-Metabolic Risk Markers. Nutr. Metab. Cardiovasc. Dis..

[28] Miller M., Beach V., Sorkin J.D., Mangano C., Dobmeier C., Novacic D., Rhyne J., Vogel R.A. (2009). Comparative Effects of Three Popular Diets on Lipids, Endothelial Function, and C-Reactive Protein during Weight Maintenance. J. Am. Diet. Assoc..

[29] Rasmussen L.G., Winning H., Savorani F., Toft H., Larsen T.M., Dragsted L.O., Astrup A., Engelsen S.B. (2012). Assessment of the Effect of High or Low Protein Diet on the Human Urine Metabolome as Measured by NMR. Nutrients.

[30] Schmedes M., Balderas C., Aadland E.K., Jacques H., Lavigne C., Graff I.E., Eng Ø., Holthe A., Mellgren G., Young J.F. (2018). The Effect of Lean-Seafood and Non-Seafood Diets on Fasting and Postprandial Serum Metabolites and Lipid Species: Results from a Randomized Crossover Intervention Study in Healthy Adults. Nutrients.

[31] Stella C., Beckwith-Hall B., Cloarec O., Holmes E., Lindon J.C., Powell J., Van Der Ouderaa F., Bingham S., Cross A.J., Nicholson J.K. (2006). Susceptibility of Human Metabolic Phenotypes to Dietary Modulation. J. Proteome Res..

[32] Tate B.N., Van Guilder G.P., Aly M., Spence L.A., Diaz-Rubio M.E., Le H.H., Johnson E.L., McFadden J.W., Perry C.A. (2023). Changes in Choline Metabolites and Ceramides in Response to a DASH-Style Diet in Older Adults. Nutrients.

[33] Genoni A., Lo J., Lyons-Wall P., Boyce M.C., Christophersen C.T., Bird A., Devine A. (2019). A Paleolithic Diet Lowers Resistant Starch Intake but Does Not Affect Serum Trimethylamine-N-Oxide Concentrations in Healthy Women. Br. J. Nutr..

[34] Griffin L.E., Djuric Z., Angiletta C.J., Mitchell C.M., Baugh M.E., Davy K.P., Neilson A.P. (2019). A Mediterranean Diet Does Not Alter Plasma Trimethylamine N-Oxide Concentrations in Healthy Adults at Risk for Colon Cancer. Food Funct..

[35] Sacks F.M., Bray G.A., Carey V.J., Smith S.R., Ryan D.H., Anton S.D., McManus K., Champagne C.M., Bishop L.M., Laranjo N. (2009). Comparison of Weight-Loss Diets with Different Compositions of Fat, Protein, and Carbohydrates. N. Engl. J. Med..

[36] Heianza Y., Sun D., Smith S.R., Bray G.A., Sacks F.M., Qi L. (2018). Changes in Gut Microbiota-Related Metabolites and Long-Term Successful Weight Loss in Response to Weight-Loss Diets: The POUNDS Lost Trial. Diabetes Care.

[37] Videja M., Sevostjanovs E., Upmale-Engela S., Liepinsh E., Konrade I., Dambrova M. (2022). Fasting-Mimicking Diet Reduces Trimethylamine N-Oxide Levels and Improves Serum Biochemical Parameters in Healthy Volunteers. Nutrients.

[38] Wiese G.N., Biruete A., Stremke E.R., Lindemann S.R., Jannasch A., Moorthi R.N., Moe S.M., Swanson K.S., Cross T.W., Hill Gallant K.M. (2024). Gut Microbiota and Uremic Retention Solutes in Adults With Moderate CKD: A 6-Day Controlled Feeding Study. J. Ren. Nutr..

[39] Ahrens A.P., Culpepper T., Saldivar B., Anton S., Stoll S., Handberg E.M., Xu K., Pepine C., Triplett E.W., Aggarwal M. (2021). A Six-Day, Lifestyle-Based Immersion Program Mitigates Cardiovascular Risk Factors and Induces Shifts in Gut Microbiota, Specifically Lachnospiraceae, Ruminococcaceae, Faecalibacterium Prausnitzii: A Pilot Study. Nutrients.

[40] Battillo D.J., Malin S.K. (2023). Impact of Caloric Restriction and Exercise on Trimethylamine N-Oxide Metabolism in Women with Obesity. Nutrients.

[41] Erickson M.L., Malin S.K., Wang Z., Mark Brown J., Hazen S.L., Kirwan J.P. (2019). Effects of Lifestyle Intervention on Plasma Trimethylamine N-Oxide in Obese Adults. Nutrients.

[42] Steele C.N., Baugh M.E., Griffin L.E., Neilson A.P., Davy B.M., Hulver M.W., Davy K.P. (2021). Fasting and Postprandial Trimethylamine N-Oxide in Sedentary and Endurance-Trained Males Following a Short-Term High-Fat Diet. Physiol. Rep..

[43] Guedes M.R., da Silva Pontes K.S., Valença D.C.T., Oigman W., Neves M.F., Klein M.R.S.T. (2020). Rationale and Design of a Randomized Controlled Trial to Evaluate the Effects of Probiotics during Energy Restriction on Blood Pressure, Body Composition, Metabolic Profile and Vascular Function in Obese Hypertensive Individuals. Artery Res..

[44] Cho C.E., Caudill M.A. (2017). Trimethylamine-N-Oxide: Friend, Foe, or Simply Caught in the Cross-Fire?. Trends Endocrinol. Metab..

[45] Nowiński A., Ufnal M. (2018). Trimethylamine N-Oxide: A Harmful, Protective or Diagnostic Marker in Lifestyle Diseases?. Nutrition.

[46] Zeisel S.H., Warrier M. (2017). Trimethylamine N-Oxide, the Microbiome, and Heart and Kidney Disease. Annu. Rev. Nutr..

[47] Kohlmeier M., Da Costa K.A., Fischer L.M., Zeisel S.H. (2005). Genetic Variation of Folate-Mediated One-Carbon Transfer Pathway Predicts Susceptibility to Choline Deficiency in Humans. Proc. Natl. Acad. Sci. USA.

[48] Mohan D., Mente A., Dehghan M., Rangarajan S., O’Donnell M., Hu W., Dagenais G., Wielgosz A., Lear S., Wei L. (2021). Associations of Fish Consumption With Risk of Cardiovascular Disease and Mortality Among Individuals With or Without Vascular Disease From 58 Countries. JAMA Intern. Med..

[49] Drouin-Chartier J.P., Chen S., Li Y., Schwab A.L., Stampfer M.J., Sacks F.M., Rosner B., Willett W.C., Hu F.B., Bhupathiraju S.N. (2020). Egg Consumption and Risk of Cardiovascular Disease: Three Large Prospective US Cohort Studies, Systematic Review, and Updated Meta-Analysis. BMJ.

[50] Wiese G.N., Biruete A., Moorthi R.N., Moe S.M., Lindemann S.R., Hill Gallant K.M. (2021). Plant-Based Diets, the Gut Microbiota, and Trimethylamine N-Oxide Production in Chronic Kidney Disease: Therapeutic Potential and Methodological Considerations. J. Ren. Nutr..

[51] O’Sullivan O., Cronin O., Clarke S.F., Murphy E.F., Molloy M.G., Shanahan F., Cotter P.D. (2015). Exercise and the Microbiota. Gut Microbes.

[52] Chen J., Guo Y., Gui Y., Xu D. (2018). Physical Exercise, Gut, Gut Microbiota, and Atherosclerotic Cardiovascular Diseases. Lipids Health Dis..

[53] Bird S.R., Hawley J.A. (2017). Update on the Effects of Physical Activity on Insulin Sensitivity in Humans. BMJ Open Sport. Exerc. Med..

[54] Gleeson M., Bishop N.C., Stensel D.J., Lindley M.R., Mastana S.S., Nimmo M.A. (2011). The Anti-Inflammatory Effects of Exercise: Mechanisms and Implications for the Prevention and Treatment of Disease. Nat. Rev. Immunol..

[55] Boulangé C.L., Neves A.L., Chilloux J., Nicholson J.K., Dumas M.E. (2016). Impact of the Gut Microbiota on Inflammation, Obesity, and Metabolic Disease. Genome Med..

[56] Yu D., Shu X.O., Rivera E.S., Zhang X., Cai Q., Calcutt M.W., Xiang Y.B., Li H., Gao Y.T., Wang T.J. (2019). Urinary Levels of Trimethylamine-N-Oxide and Incident Coronary Heart Disease: A Prospective Investigation Among Urban Chinese Adults. J. Am. Heart Assoc..

[57] Han J.M., Guo L., Chen X.H., Xie Q., Song X.Y., Ma Y.L. (2024). Relationship between Trimethylamine N-Oxide and the Risk of Hypertension in Patients with Cardiovascular Disease: A Meta-Analysis and Dose-Response Relationship Analysis. Medicine.

[58] Kühn T., Rohrmann S., Sookthai D., Johnson T., Katzke V., Kaaks R., Von Eckardstein A., Müller D. (2017). Intra-Individual Variation of Plasma Trimethylamine-N-Oxide (TMAO), Betaine and Choline over 1 Year. Clin. Chem. Lab. Med..

[59] Bradley E., Haran J. (2024). The Human Gut Microbiome and Aging. Gut Microbes.

[60] Yoon K., Kim N. (2021). Roles of Sex Hormones and Gender in the Gut Microbiota. J. Neurogastroenterol. Motil..

[61] Brooks A.W., Priya S., Blekhman R., Bordenstein S.R. (2018). Gut Microbiota Diversity across Ethnicities in the United States. PLoS Biol..

[62] Andrikopoulos P., Aron-Wisnewsky J., Chakaroun R., Myridakis A., Forslund S.K., Nielsen T., Adriouch S., Holmes B., Chilloux J., Vieira-Silva S. (2023). Evidence of a Causal and Modifiable Relationship between Kidney Function and Circulating Trimethylamine N-Oxide. Nat. Commun..

[63] Missailidis C., Hällqvist J., Qureshi A.R., Barany P., Heimbürger O., Lindholm B., Stenvinkel P., Bergman P. (2016). Serum Trimethylamine-N-Oxide Is Strongly Related to Renal Function and Predicts Outcome in Chronic Kidney Disease. PLoS ONE.

