# Supplementary material for: The Association Between Lifestyle Interventions and Trimethylamine N-Oxide: A Systematic-Narrative Hybrid Literature Review
Source: Nutrients. 2025 Apr 6;17(7):1280. doi: 10.3390/nu17071280 (PMC11990624; doi:10.3390/nu17071280)
Supplement: Supplementary file 1 [file nutrients-17-01280-s001.zip › nutrients-3530353-supplementary.pdf]

Contents

Supplementary Figure S1. Flowchart of the eligibility process

Supplementary Table S1. Search strategy for identifying studies on PubMed

Supplementary Figure S1. Flowchart of the eligibility process

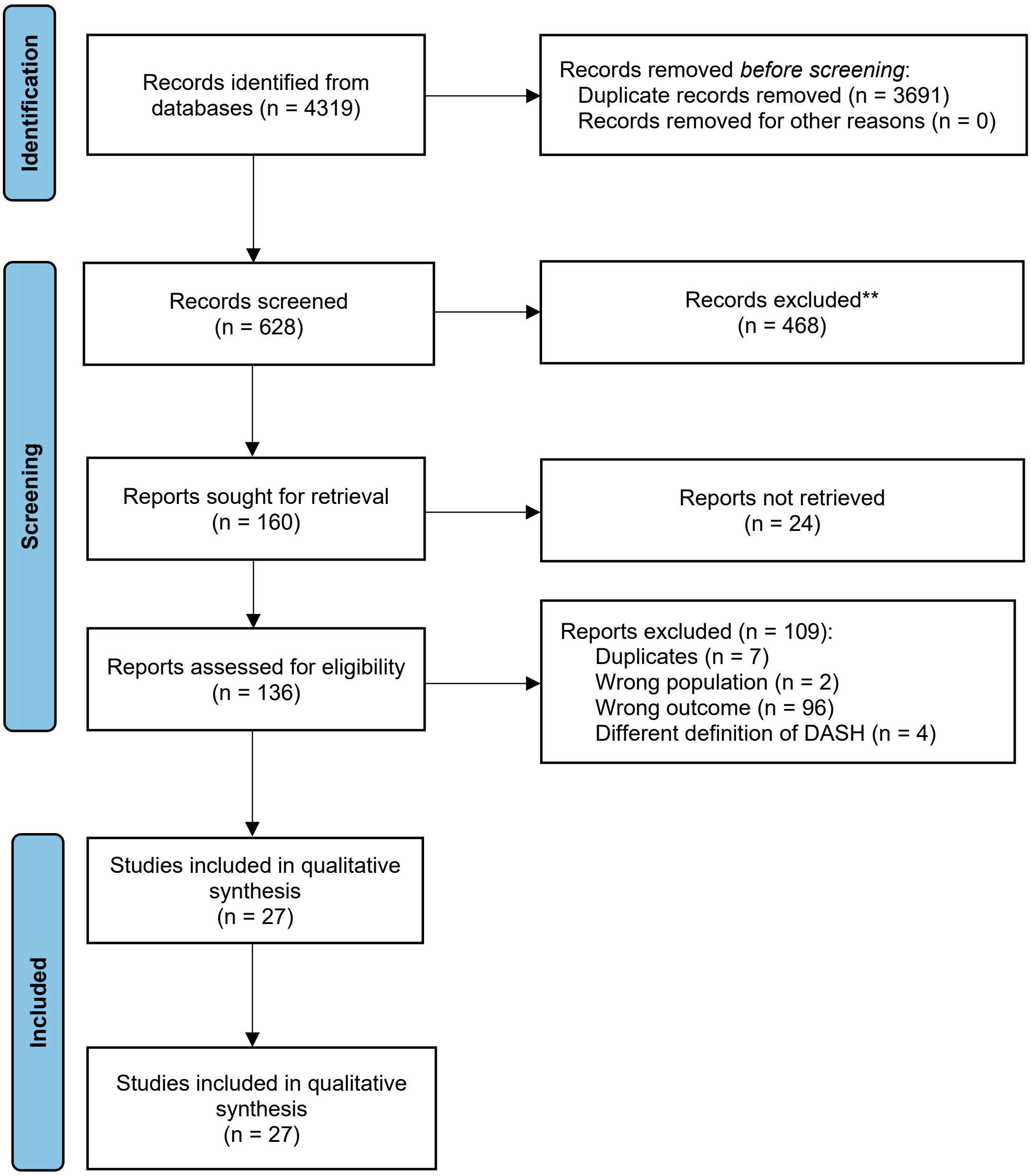

Supplementary Table S1. Search strategy for identifying studies on PubMed.

**Search**	Query
#1	TMAO [Title/Abstract]
#2	trimethylamine N-oxide [Title/Abstract]
#3	1-2/OR
#4	lifestyle intervention* [Title/Abstract]
#5	diet [MeSH Terms]
#6	diet* [Title/Abstract]
#7	physical activity [Title/Abstract]
#8	exercise[Title/Abstract]
#9	exercise[MeSH Terms]
#10	4-9/OR
#11	#3 AND #10
The last search performed in July 2024	
